# Emotional labor and professional identity among pre-service early childhood teachers: the mediating role of emotional exhaustion and the moderating role of social and emotional competence

**DOI:** 10.3389/fpsyg.2026.1830373

**Published:** 2026-05-25

**Authors:** Yuanyuan Liu, Zhongyi Xin

**Affiliations:** 1 Faculty of Preschool Education, Shaanxi Xueqian Normal University, Xi’an, China; 2 Faculty of Education Science, Shaanxi Xueqian Normal University, Xi’an, China

**Keywords:** emotional exhaustion, emotional labor, pre-service early childhood teachers, professional identity, social and emotional competence

## Abstract

**Objective:**

This study examined the influence of emotional labor on professional identity among pre-service early childhood teachers, exploring the mediating role of emotional exhaustion and the moderating role of Social and Emotional Competence.

**Methods:**

A survey was conducted with 654 pre-service early childhood educators. Participants completed measures of emotional labor, emotional exhaustion, social and emotional competence, and professional identity. A moderated mediation model was analyzed using SPSS PROCESS Macro.

**Results:**

Mediation analysis indicated that emotional exhaustion partially mediated the relationship between different dimensions of emotional labor and professional identity. Social and emotional competence (SEC) negatively moderated the direct relationship between surface acting (SA) and emotional exhaustion; However, this moderating effect was not significant for deep acting (DA) or expression of naturally felt emotions (ENFE). Individuals with lower levels of SEC exhibited a stronger positive relationship between SA and emotional exhaustion; as SEC increased, this relationship became less significant.

**Conclusion:**

This study advances the understanding of how different emotional labor strategies influence professional identity among pre-service early childhood teachers. It identifies a suppression effect for SA and demonstrates that SEC functions as a moderator only for SA. The results highlight the importance of enhancing pre-service teachers’ SEC to mitigate the resource-depleting effects of surface acting. This study contributes to the literature on teacher development and provides theoretical implications for fostering professional identity in early childhood education.

## 1 Introduction

The development and cultivation of professional identity among pre-service teachers have increasingly become focal points for educational researchers and practitioners. As a reserve force for the future teaching workforce, the professional identity of pre-service teachers (PSTs) not only directly influences their learning engagement and career choice intentions but is also crucial for the stability of the future teaching workforce and the improvement of educational quality (Hong, 2010; Liu and Boyd, 2018; Wang et al., 2020; Sun et al., 2025). Previous studies have explored the factors influencing the professional identity of pre-service teachers from multiple perspectives, including demographic characteristics (e.g., gender and grade level) (Xie et al., 2022; Saied and Rusu, 2023), situational factors (e.g., university coursework, type of educators, educational models, and teaching internship) (Chen and Mensah, 2018; Cai et al., 2022), and individual factors (e.g., emotional experiences, self-efficacy, and prior experiences) (Izadinia, 2012; Chen et al., 2020; Chen et al., 2022). Among these various influencing factors, emotions are regarded as a core factor affecting the professional identity of pre-service teachers (Beauchamp and Thomas, 2009; Rodrigues and Mogarro, 2019). In particular, during teaching internships, the complex emotional experiences encountered by pre-service teachers play a critical role in the formation and development of their professional identity (Timoštšuk and Ugaste, 2012; Zhu, 2017; Draves, 2020).

Despite the significant foundation that existing research provides for understanding pre-service teachers’ professional identity, emotional labor as a strategy for proactive emotion management and regulation has received little attention. However, its direct and indirect mechanisms of influence on professional identity remain unclear. In particular, how emotional exhaustion, a widely studied consequence of emotional labor, mediates this process and which personal resources (e.g., social and emotional competence) can buffer this impact have yet to be empirically examined.

The high emotional labor characteristic of pre-service teachers in early childhood education during internships makes them an ideal sample for examining these mechanisms. Based on this, the present study focuses on pre-service early childhood education teachers, integrating job demands-resources (JD-R) theory (Bakker and Demerouti, 2017) and conservation of resources (COR) theory (Hobfoll, 2001) to construct a moderated mediation model. This model explores the impact of emotional labor on professional identity, alongside the mediating roles of emotional exhaustion and social and emotional competence (SEC).

### 1.1 The relationship between emotional labor and professional identity

To comply with professional norms and meet job-related emotional requirements, teachers need to regulate their emotions, thereby giving rise to emotional labor (Grandey, 2000), which permeates teachers’ various professional responsibilities and interactions (Brown et al., 2023). The attribute of emotional labor is particularly pronounced in the field of early childhood education (Carey and Sutton, 2024). Since Hochschild first explicitly defined emotional labor in 1983, this field has garnered extensive attention from researchers. Research on emotional labor strategies has gradually expanded from the two strategies of surface acting and deep acting to three strategies encompassing surface acting (SA), deep acting (DA), and expression of naturally felt emotions (ENFE) (Diefendorff et al., 2005; Grandey and Melloy, 2017; Wang et al., 2019). SA refers to individuals hiding their true inner feelings and feigning emotional expressions that meet job requirements; DA refers to individuals actively adjusting their inner feelings to align their genuine emotions with required expressions; ENFE refers to a state in which the emotions individuals actually experience naturally correspond with job requirements, requiring no additional regulatory effort. Numerous studies have demonstrated that teachers’ emotional labor profoundly influences their professional efficacy, job satisfaction, and personal well-being (Grandey, 2000; Yin et al., 2019; Wang et al., 2019; Wang et al., 2023).

Researchers consistently acknowledge that emotions are crucial factors influencing pre-service teachers’ professional identity (Teng, 2017; Zembylas, 2003; Rodrigues and Mogarro, 2019; Savina et al., 2025), and emotional labor serves as an important pathway for shaping professional identity (Deng et al., 2018). However, the relationship between emotional labor and professional identity is complex. Following social identity theory, emotional labor is an active process through which employees construct their role identity, rather than a purely external imposition. Emotional labor is moderated by one’s social and personal identities and stimulates pressures for the person to identify with the service role (Ashforth and Humphrey, 1993). Findings from available research remain inconclusive on whether emotional labor undermines (Oh, 2022) or promotes (Nazari and Karimpour, 2022; Nazari et al., 2023) professional identity. This phenomenon may reflect the dual nature of emotional labor. Specifically, Engaging in emotional labor, particularly through deep acting or genuine expression, enables pre-service teachers to internalize professional display rules. Such experiences form a foundational pathway for constructing professional identity. Conversely, sustained emotional labor (especially SA) depletes psychological resources. This process may generate negative outcomes that hinder identity formation. This suggests to researchers that more complex mediating and moderating mechanisms may exist between emotional labor and professional identity, thereby influencing the direction and strength of their relationship.

### 1.2 The mediating role of emotional exhaustion

According to the Conservation of Resources (COR) theory (Hobfoll, 2001) and the Job Demands-Resources (JD-R) theory (Bakker and Demerouti, 2017), emotional labor, as an affective job demand, consumes individuals’ limited psychological resources. When resource consumption exceeds replenishment, it leads to consequences such as emotional exhaustion (Brotheridge and Lee, 2002) and may result in broader resource loss, including diminished professional identity, due to insufficient resources to sustain positive occupational engagement. Crucially, Hobfoll et al. (2018) explicitly note that “COR theory is largely the basis for the more work-specific leading theory of organizational stress, namely the job demands-resources model.” These two theories are complementary in the present study: the JD-R theory conceptualizes emotional labor as a job demand, while the COR theory explains why job demands trigger resource depletion and how such depletion leads to negative outcomes. Together, they provide a robust theoretical foundation for investigating the mechanisms through which emotional labor influences professional identity.

Currently, emotional exhaustion is widely recognized as a significant outcome variable of emotional labor (Judge et al., 2009; Grandey and Gabriel, 2015; Grandey and Melloy, 2017; Jeung et al., 2018; Zhang et al., 2020; Kariou et al., 2021; Lampert and Hornung, 2025). Emotional exhaustion is primarily characterized by a lack of vitality, representing a sense of being depleted of emotional resources, and serves as the most indicative dimension of occupational burnout (Maslach et al., 2001; Chang, 2009; Keller et al., 2014). As a typical state indicating resource depletion, emotional exhaustion weakens individuals’ affective commitment and value identification with their professional roles. Existing research has found that emotional exhaustion is negatively associated with positive occupational outcomes, such as teachers’ professional commitment and job satisfaction (Chen et al., 2024; Lee, 2019). In light of these analyses, this study hypothesizes that emotional exhaustion mediates the relationship between emotional labor and professional identity.

### 1.3 The moderating role of social and emotional competence

The impact of emotional labor on emotional exhaustion has produced inconsistent research findings. Meta-analytic research on emotional labor indicates that different strategy dimensions exhibit significant differences in outcome variables (Wang et al., 2019; Humphrey, 2023). A significant body of research indicates that SA significantly and positively predicts emotional exhaustion. However, the relationships between the other two strategies (DA and ENFE) and emotional exhaustion vary considerably across studies. Specifically, DA has been found to be positively correlated (Grandey and Melloy, 2017), negatively correlated (Hsu, 2017; Moon et al., 2013), and uncorrelated (Zhang et al., 2020) with emotional exhaustion; ENFE has been found to be negatively correlated (Lee et al., 2019) and uncorrelated (Ma et al., 2023) with emotional exhaustion. According to COR theory, this inconsistency may be attributable to differences in individuals’ levels of personal resources. Those with higher resources, such as social–emotional competence, are better able to resist the resource depletion caused by job demands and to interrupt the resource loss spiral through resource investment, thereby altering the strength or direction of the relationship between emotional labor and emotional exhaustion. Thus, this inconsistency suggests the possible existence of contextual variables that moderate the relationship between the two.

In recent years, SEC has been recognized as a crucial personal psychological resource. It is considered an important protective factor that helps teachers more effectively cope with professional challenges and serves as a key indicator influencing educational quality (Buettner et al., 2016; Oliveira et al., 2021; Nwoko et al., 2023). SEC encompasses various dimensions, such as self-awareness, self-management, social awareness, relationship skills, and responsible decision-making (Collaborative for Academic, Social, and Emotional Learning, 2022), providing individuals with diverse emotion regulation strategies. According to emotion regulation theory (Gross, 2015), individuals’ emotion regulation abilities influence the effectiveness of strategy selection and implementation. As an integrated form of emotion regulation ability, SEC allows individuals to select and execute adaptive strategies, thus mitigating the resource depletion associated with emotional labor. Research has confirmed that individual differences (e.g., emotional intelligence and personality) can moderate the effects of emotional labor (Judge et al., 2009; Kiffin-Petersen et al., 2011; Grandey and Gabriel, 2015; Grandey and Melloy, 2017). Based on COR theory, social–emotional competence, as an important stock of personal resources, can interrupt the resource loss spiral triggered by surface acting through resource investment. Moreover, according to the person × situation interaction perspective of JD-R theory (Bakker et al., 2023), the detrimental effect of a job demand on emotional exhaustion depends on the level of personal resources available to the individual. Therefore, the present study introduces social–emotional competence as a moderator to examine its buffering role in the relationship between emotional labor and emotional exhaustion.

### 1.4 The present study

Despite extensive research on the relationship between emotional labor and professional identity among in-service teachers, little is known about how different dimensions of emotional labor influence professional identity in pre-service early childhood teachers, particularly regarding the mediating role of emotional exhaustion and the moderating role of SEC. To address this gap, this study is guided by three key research questions: Do SA, DA, and ENFE have differential effects on pre-service teachers’ professional identity? Does emotional exhaustion mediate the relationships between each dimension of emotional labor and professional identity? Does SEC moderate the relationship between each dimension of emotional labor and emotional exhaustion?

**Figure 1:**
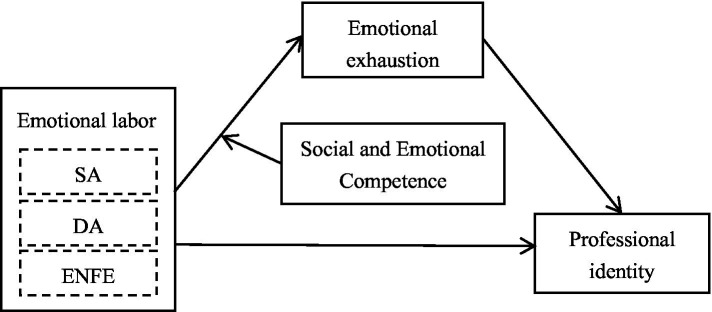
The hypothesized research model.

Based on the theoretical framework and empirical evidence, this study focuses on pre-service early childhood teachers in China and develops a moderated mediation model (Figure 1) to examine how emotional labor impacts professional identity. Specifically, emotional labor is conceptualized as three distinct strategies (surface acting, deep acting, expression of naturally felt emotions), all of which are hypothesized to influence professional identity through the mediating role of emotional exhaustion. Drawing on the JD-R theory and the COR theory, emotional labor (especially SA) is viewed as a job demand that depletes psychological resources, leading to emotional exhaustion, which in turn reduces professional identity. Social and emotional competence is proposed as a moderator on the path from emotional labor to emotional exhaustion, such that higher SEC buffers the resource depletion effect by interrupting the loss spiral. The following hypotheses are proposed:

*H1*: Different dimensions of emotional labor have differential effects on PSTs’ professional identity.

*H1a*: DA positively predicts PSTs’ professional identity.

*H1b*: ENFE positively predicts PSTs’ professional identity.

*H1c*: SA does not exhibit positive total effect on PSTs’ professional identity.

*H2*: Emotional exhaustion mediates the relationship between emotional labor and professional identity.

*H3*: Social and emotional competence negatively moderates the relationship between emotional labor and emotional exhaustion.

## 2 Method

### 2.1 Participants

The study involved pre-service early childhood education (ECE) majors from six universities in Shaanxi Province, China. In China, PSTs participate in two types of educational practices: educational observation and teaching internship. Educational observation is a short-term practice spread across 4 years of university, whereas teaching internship is a concentrated activity typically conducted in the final year. We collected data after the participants completed their annual educational observations or teaching internships. By collaborating with university faculty members, we distributed questionnaire links to the target participants through the online survey platform Questionnaire Star. Participants were fully informed about the study’s purpose and voluntarily completed the questionnaires within the specified timeframe. Owing to the gender imbalance in ECE, we specifically invited male participants to enhance sample representativeness.

A total of 709 questionnaires were collected. After excluding invalid questionnaires, including those with patterned responses and incorrect choices on attitude test items, 654 valid questionnaires were obtained. The valid response rate was 92.2%. Of the valid sample, 147 participants (22.5%) were men and 507 were women (77.5%). Regarding grade level, 141 participants (21.6%) were sophomores, 348 (53.2%) were juniors, and 165 (25.2%) were seniors. In terms of institutional type, 494 participants (75.5%) came from local normal universities, 65 (10.0%) from comprehensive universities, and 95 (14.5%) from higher vocational colleges. Concerning internship experience, 136 participants (20.8%) had completed one formal internship, 118 (18.0%) two, 110 (16.8%) three, and 290 (44.3%) had four or more internships.

### 2.2 Measures

#### 2.2.1 Emotional labor scale (ELS)

Emotional labor was assessed using the Emotional Labor Scale developed by Diefendorff et al. (2005) and validated in the Chinese context by Lu (2023). The scale contains 13 items across three dimensions: surface acting, deep acting, and expression of naturally felt emotions. Items were rated on a 5-point Likert scale from 1 (strongly disagree) to 5 (strongly agree). Higher scores indicate a stronger tendency to adopt specific emotional labor strategies to meet work-role requirements. In this study, the internal consistency of the Emotional Labor Scale was assessed using Cronbach’s *α* coefficient. The overall scale demonstrated acceptable internal consistency (*α* = 0.768). For the subscales, surface acting (*α* = 0.781), deep acting (*α* = 0.769), and ENFE (*α* = 0.714) all exceeded the conventional reliability threshold, indicating acceptable internal consistency.

#### 2.2.2 Emotional exhaustion scale (EES)

Emotional exhaustion was measured using the emotional exhaustion subscale of the Maslach Burnout Inventory-General Survey (MBI-GS) (Schaufeli et al., 1996), revised by Li and Shi (2003) for the Chinese cultural context. It includes five items rated on a 5-point Likert scale from 1 (strongly disagree) to 5 (strongly agree). Higher scores suggest greater emotional exhaustion. The Cronbach’s *α* coefficient for this scale in our study was 0.933, indicating excellent internal consistency.

#### 2.2.3 Professional identity scale of pre-service teacher (PISPT)

Professional identity was assessed using the Pre-service Teacher Professional Identity Scale developed by Zhao et al. (2012). It comprises 15 items across three dimensions: intrinsic value identification, extrinsic value identification, and volitional behavior identification. Items are rated on a 5-point Likert scale from 1 (strongly disagree) to 5 (strongly agree), with higher scores reflecting higher professional identity levels. The Cronbach’s α coefficient for our study was 0.941, reflecting excellent internal consistency among the items.

#### 2.2.4 Social and emotional competence scale (SECS)

We measured SEC using the College Students’ Social and Emotional Competence Scale developed by Chen et al. (2023). The scale encompasses 26 items across four dimensions: self-relationship, other-relationship, collective-relationship, and responsible decision-making abilities. Items were rated on a 5-point Likert scale from 1 (strongly disagree) to 5 (strongly agree), with higher scores indicating elevated levels of SEC. The Cronbach’s α coefficient for this scale was 0.965 in our study, which demonstrates excellent internal consistency.

### 2.3 Data analysis

Data were collected using the online survey platform Questionnaire Star. For preliminary analyses, SPSS 29.0 was employed to conduct Cronbach’s α reliability tests, descriptive statistics, and Pearson correlations among the main variables. SPSS (EFA) and Mplus 8.3 were used to conduct the common method bias test. The mediating role of emotional exhaustion was tested using PROCESS Model 4 (Hayes, 2018) with 5,000 bootstrap samples. To test the moderating role of social and emotional competence (SEC) and the overall moderated mediation model, we performed a moderated mediation analysis using PROCESS Model 7 with 5,000 bootstrap samples to generate 95% bias-corrected confidence intervals for conditional indirect effects at low, mean, and high levels of SEC.

## 3 Results

### 3.1 Test of common method bias

Given that this study employed self-report questionnaires, common method bias may be a potential concern. To minimize this risk, both procedural and statistical remedies were adopted. Differences in response formats were applied across the questionnaires: some scales were rated from 1 (“strongly disagree”) to 5 (“strongly agree”), whereas others ranged from 1 (“never”) to 5 (“always”). All questionnaires were completed anonymously to reduce evaluation concerns, and demographic questions were placed at the end of the survey. During the data analysis phase, Harman’s single-factor test was conducted by performing an exploratory factor analysis on all measurement items. The results revealed nine factors with eigenvalues greater than 1, with the first factor accounting for 32.824% of the total variance. This value is below the critical threshold of 40% (Podsakoff et al., 2003; Podsakoff et al., 2024). Subsequently, a confirmatory factor analysis (CFA) was performed using Mplus 8.3 to test the single-factor model. The fit indices indicated poor model fit (*χ*^2^/df = 6.446, CFI = 0.482, TLI = 0.463, RMSEA = 0.129, SRMR = 0.127). Taken together, these findings suggest that common method variance was effectively controlled to a certain degree in this study.

### 3.2 Correlation analysis

Correlation analyses were conducted to examine the relationships among emotional labor, emotional exhaustion, professional identity, and SEC in pre-service early childhood teachers during their observation/internship period (Table 1). Correlation analyses indicated that professional identity was not significantly correlated with SA but was significantly positively correlated with DA, ENFE, SEC, and significantly negatively correlated with emotional exhaustion. SEC was not significantly correlated with SA, but was significantly positively correlated with DA and ENFE, and significantly negatively correlated with emotional exhaustion. Emotional exhaustion was significantly positively correlated with SA and significantly negatively correlated with DA and ENFE.

**Table 1:** The correlations among study variables.

Variables	*M*	SD	1	2	3	4	5	6
1. SA	3.384	0.719	1					
2. DA	4.110	0.524	0.124**	1				
3. ENFE	4.034	0.566	0.089*	0.673**	1			
4. EE	3.051	0.827	0.186**	−0.183**	−0.157**	1		
5. SEC	4.200	0.417	−0.032	0.503**	0.439**	−0.123**	1	
6. PI	3.859	0.584	0.019	0.444**	0.422**	−0.297**	0.496**	1

*p < 0.05, **p < 0.01; SA, surface acting; DA, deep acting; ENFE, expression of naturally felt emotions; EE, emotional exhaustion; PI, professional identity.

### 3.3 Mediation analysis

To explore the mediating effect of emotional exhaustion in the relationship between various dimensions of emotional labor and professional identity, a mediation analysis was conducted using Model 4 of the PROCESS macro for SPSS. Gender and grade level were included as covariates. SA, DA, and ENFE were considered independent variables, emotional exhaustion as the mediator, and professional identity as the dependent variable (Table 2).

**Table 2:** Mediation model effects.

Predictor	Path	*β*	*t*	*p*	95% CI
SA	SA → EE	0.185	4.837	<0.001	[0.127, 0.299]
	EE → PI	−0.309	−8.041	<0.001	[−0.271, −0.165]
	Direct effect (SA → PI)	0.077	2.011	0.045	[0.001, 0.123]
	Total effect (SA → PI)	0.020	0.497	0.619	[−0.047, 0.078]
	Indirect effect (SA → EE → PI)	−0.057	-	-	[−0.087, −0.030]
DA	DA → EE	−0.188	−4.917	<0.001	[−0.415, −0.178]
	EE → PI	−0.217	−6.190	<0.001	[−0.201, −0.104]
	Direct effect (DA → PI)	0.407	11.689	<0.001	[0.377, 0.529]
	Total effect (DA → PI)	0.447	12.738	<0.001	[0.421, 0.575]
	Indirect effect (DA → EE → PI)	0.041	-	-	[0.016, 0.071]
ENFE	ENFE → EE	−0.169	−4.349	<0.001	[−0.358, −0.135]
	EE → PI	−0.228	−6.492	<0.001	[−0.209, −0.112]
	Direct effect (ENFE→PI)	0.395	11.229	<0.001	[0.336, 0.479]
	Total effect (ENFE→PI)	0.434	12.121	<0.001	[0.375, 0.520]
	Indirect effect (ENFE→EE → PI)	0.038	-	-	[0.014, 0.068]

SA, surface acting; DA, deep acting; ENFE, expression of naturally felt emotions; EE, emotional exhaustion; PI, professional identity.

SA significantly predicted emotional exhaustion (*β* = 0.185, *p* < 0.001), which in turn negatively predicted professional identity (*β* = −0.309, *p* < 0.001). The direct effect of surface acting on professional identity was also statistically significant but very small in magnitude (*β* = 0.077, *p* = 0.045). While both the direct effect (*β* = 0.077) and the indirect effect through emotional exhaustion (*β* = −0.057, 95% CI [−0.087, −0.030]) were significant, the total effect was non-significant (*β* = 0.020, *p* = 0.619, 95% CI [−0.047, 0.078]). This pattern of opposite-signed direct and indirect effects with a non-significant total effect indicates a suppression effect (Wen and Ye, 2014). The positive direct influence of surface acting on professional identity is masked by its negative indirect influence through emotional exhaustion. When these two opposing pathways cancel each other out, the total effect becomes non-significant. Thus, instead of a traditional mediation where the indirect pathway simply transmits the effect, the present results reveal a suppression effect.

The DA significantly and negatively predicts emotional exhaustion (*β* = −0.188, *t* = −4.917, *p* < 0.001), representing a small-to-moderate effect size; this aligns with COR theory’s gain spiral, as deep acting may replenish rather than deplete emotional resources. Emotional exhaustion significantly and negatively affects professional identity (*β* = −0.217, *t* = −6.190, *p* < 0.001). The direct effect of deep acting on professional identity was significant (*β* = −0.407, *t* = 11.689, *p* < 0.001), indicating a moderate-to-strong direct effect, as was the total effect (*β* = 0.447, *t* = 12.738, *p* < 0.001). Additionally, the indirect effect through emotional exhaustion was significant (*β* = 0.041, 95% CI [0.016, 0.071]), suggesting a small indirect effect. These results suggest emotional exhaustion partially mediates the relationship between deep acting and professional identity.

The ENFE significantly and negatively predicted emotional exhaustion (*β* = −0.169, *t* = −4.349, *p* < 0.001), representing a small-to-moderate effect size; this is consistent with the JD-R theory’s health impairment pathway, as ENFE imposes minimal emotional demand and thus preserves rather than depletes resources. Emotional exhaustion significantly and negatively impacting professional identity (*β* = −0.228, *t* = −6.492, *p* < 0.001). The direct effect of ENFE on professional identity was significant (*β* = 0.395, *t* = 11.229, *p* < 0.001), indicating a moderate-to-strong direct effect, as was the total effect (*β* = −0.434, *t* = 12.121, *p* < 0.001). The indirect effect through emotional exhaustion was significant (*β* = 0.038, 95% CI [0.014, 0.068]). These findings indicate that emotional exhaustion partially mediates the relationship between ENFE and professional identity.

### 3.4 Moderated mediation analysis

In this study, each dimension of emotional labor was used as the independent variable, emotional exhaustion as the mediating variable, professional identity as the dependent variable, and SEC as the moderating variable. Under the control of grade level and gender, a moderated mediation model was tested using PROCESS Model 7, with the bias-corrected percentile Bootstrap method (5,000 samples) used to assess the significance of the mediation effect. All variables were standardized prior to analysis.

#### 3.4.1 Surface acting as the independent variable

In Model 1, the main effects of surface acting (*β* = 0.185, *t* = 4.915, *p* < 0.001) and SEC (*β* = −0.160, *t* = −4.111, *p* < 0.001) on emotional exhaustion were both small-to-moderate in magnitude and significant. The interaction term between surface acting and SEC significantly predicted emotional exhaustion (*β* = −0.099, *t* = −3.632, *p* < 0.001). This indicates that SEC negatively moderates the relationship between surface acting and emotional exhaustion, consistent with COR theory’s buffering hypothesis. In Model 2, emotional exhaustion significantly and negatively predicted professional identity (*β* = −0.309, *t* = −8.041, *p* < 0.001), indicating a moderate negative effect. After controlling for emotional exhaustion, the direct effect of surface acting on professional identity remained significant (*β* = 0.077, *t* = 2.011, *p* < 0.05) (Table 3).

**Table 3:** Moderated mediation effect analysis.

Variables	Model 1 (M: EE)				Model 2 (Y: PI)			
	*β*	SE	*t*	95% CI	*β*	SE	*t*	95% CI
SA	0.185	0.038	4.915***	[0.111, 0.259]	0.077	0.038	2.011*	[0.002, 0.152]
EE	–	–	–	–	−0.309	0.038	−8.041***	[−0.384, −0.233]
SEC	−0.160	0.039	−4.111***	[−0.237, −0.084]	–	–	–	–
EL × SEC	−0.099	0.027	−3.632***	[−0.152, −0.045]	–	–	–	–
*R* ^2^	0.086				0.094			
*F*	12.247***				16.893***			

SA, surface acting; DA, deep acting; ENFE, expression of naturally felt emotions; SEC, SEC; EE, emotional exhaustion; PI, professional identity; *p < 0.05, **p < 0.01, ***p < 0.001.

To further explore the specific pattern of the moderating effect, simple slope analyses were conducted to examine the effect of surface acting on emotional exhaustion at low (*M* − 1SD) and high (*M* + 1SD) levels of SEC. The results are presented in Table 4. The index of moderated mediation was significant (Index = 0.031, SE = 0.011, 95% CI [0.008, 0.051]), providing further evidence for the presence of a moderated mediation effect, with the moderation occurring on the first stage of the mediation pathway.

**Table 4:** Conditional indirect effect results.

Variables		Effect	SE	95% CI	
SEC	M-1SD	−0.088	0.020	−0.128	−0.050
	M + 1SD	−0.027	0.017	−0.063	0.003
Index of moderated mediation		0.031	0.011	0.008	0.051

**Figure 2:**
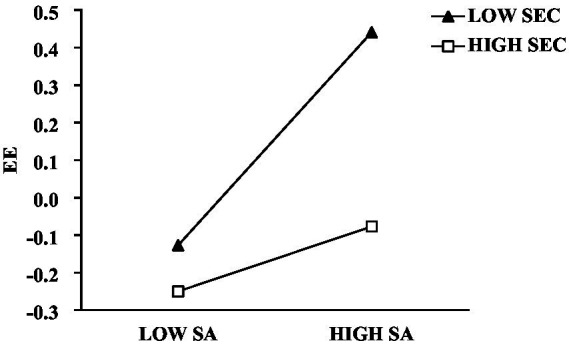
Simple slope analysis of the moderating effect of social and emotional competence on the relationship between surface acting and professional identity.

Simple slope analysis (Figure 1) revealed that when SEC was low (−1 SD), SA positively and significantly predicted emotional exhaustion (*β* = 0.284, *t* = 6.006, *p* < 0.001); however, when SEC was high (+1 SD), the predictive effect of surface acting on emotional exhaustion was no longer significant (*β* = 0.086, *t* = 1.891, *p* = 0.059). In other words, as SEC increased, the indirect effect of surface acting on professional identity through emotional exhaustion gradually diminished (Figure 2).

#### 3.4.2 Deep acting and expression of naturally felt emotions as independent variables

The model with DA as the independent variable showed that the interaction term between DA and SEC significantly predicted emotional exhaustion (*β* = −0.070, *p* < 0.001). However, the index of moderated mediation was 0.015, and its bootstrap confidence interval contained zero (95% CI [−0.004, 0.024] [−0.004, 0.024]), indicating that the indirect effect of deep acting on professional identity through emotional exhaustion did not differ significantly across levels of SEC. Thus, the moderated mediation effect was not established.

The model with ENFE as the independent variable exhibited a similar pattern: the interaction term between ENFE and SEC significantly and negatively predicted emotional exhaustion (*β* = −0.069, *p* < 0.001). However, the index of moderated mediation was 0.016, with a bootstrap confidence interval containing zero (95% CI [−0.006, 0.025]). This suggests that the indirect effect of ENFE on professional identity via emotional exhaustion does not vary significantly as a function of SEC, indicating that the moderated mediation effect is not supported.

## 4 Discussion

Based on conservation of resources (COR) theory and self-regulation executive function theory, this study developed a moderated mediation model to explore how emotional labor impacts the professional identity of pre-service early childhood teachers. Specifically, it focuses on the mediating role of emotional exhaustion and the moderating role of SEC. The findings confirmed all the proposed hypotheses, shedding light on the boundary conditions and mechanisms underlying the “double-edged sword” effect of emotional labor. These results offer valuable theoretical and empirical insights for designing interventions that promote and enhance the professional identity of pre-service teachers.

### 4.1 The influence of emotional labor on professional identity

This study found that different dimensions of emotional labor exert differential predictive effects on professional identity among pre-service early childhood teachers, providing support for Hypothesis 1. Specifically, consistent with H1a and H1b, both DA and ENFE significantly and positively predicted professional identity. In line with H1c, SA did not exhibit a clear positive total effect on professional identity. These results reveal the complex patterns through which distinct emotional labor strategies influence the formation of PSTs professional identity.

As reported in the Results section, SA exhibited a suppression effect on professional identity. Its positive direct effect was counteracted by a negative indirect effect through emotional exhaustion, resulting in a non-significant total effect. This suppression effect highlights the importance of not over-simplifying the relationship between surface acting and professional identity. Both DA and ENFE significantly and positively predict professional identity, consistent with previous research (Hang and Chen, 2021; Zeng et al., 2021; Zhao et al., 2023; Park and Kim, 2024; Liu and Li, 2026). According to the Conservation of Resources (COR) theory (Hobfoll, 2001), individuals engaged in emotion regulation within work or learning contexts may deplete psychological resources. However, the normative experience of emotional expression can simultaneously enhance their sense of identification with their professional role (Robinson and Smith-Lovin, 1999; Brown et al., 2014). PSTs constructed meaning around their emotional labor practices, attributing value to them in terms of fostering personal development and promoting student growth (Zhang and Liu, 2024). Their emotional expression practices during teaching internships fundamentally represent an identity construction process of “becoming a teacher through action” (He et al., 2022).

These findings have important implications for pre-service teacher education. First, emotional labor can be both harmful and beneficial (Humphrey, 2023). As a form of “authentic professional experience,” emotional labor should be perceived more as a pathway for professional growth rather than a mere burden. Second, efforts should be made to help pre-service teachers recognize the differential consequences of various emotional labor strategies. They can learn to reduce their reliance on surface acting, and transform emotional labor experiences into valuable resources for professional identity development through reflective practice.

### 4.2 The mediating role of emotional exhaustion

The present study found that emotional exhaustion partially mediated the relationships between each dimension of emotional labor and professional identity, thereby supporting Hypothesis 2. However, a notable divergence emerged in the direction of these mediating effects: SA undermined professional identity by increasing emotional exhaustion, whereas DA and ENFE enhanced professional identity by reducing emotional exhaustion. These findings align closely with the tenets of Conservation of Resources (COR) theory (Hobfoll, 2001) and Job Demands-Resources (JD-R) model (Bakker and Demerouti, 2017). Specifically, SA, as a strategy characterized by emotional dissonance, extensively depletes individuals’ psychological resources, thereby predisposing them to emotional exhaustion. In contrast, DA and ENFE, by achieving internal emotional alignment or requiring no additional regulatory effort, serve to protect or even augment psychological resources (Gross and John, 2003; Richards and Gross, 2000).

For SA, the results showed a significant positive direct effect on professional identity and a significant negative indirect effect through emotional exhaustion, with the total effect being non-significant. This is a pattern indicative of a suppression effect. Specifically, SA significantly and positively predicted emotional exhaustion, which in turn negatively influenced professional identity. This positive association between SA and emotional exhaustion is one of the most robust findings in the literature, consistent with the meta-analytic evidence from Wang et al. (2019) based on in-service teachers, as well as the review conclusions of Purper et al. (2023) focusing on early childhood educators. By expanding the research focus to include pre-service early childhood teachers, this study corroborates the cross-situational stability of the resource-depleting effects associated with SA. Moreover, the suppression effect reveals a dual role of SA. On one hand, the significant positive direct effect may reflect superficial compliance with professional display rules. By simply “putting on a mask,” pre-service teachers meet immediate role expectations without deep emotional investment, which may temporarily enhance their perceived professional role adaptation. On the other hand, the significant negative indirect effect through emotional exhaustion captures the hidden cost of SA. Suppressing genuine emotions and faking appropriate displays require continuous self-monitoring and emotional regulation, which deplete limited psychological resources (Gross, 1998; Grandey, 2000). This sustained self-monitoring consumes cognitive resources without altering underlying negative emotional states, thereby precipitating emotional exhaustion and subsequently diminishing individuals’ affective commitment to and value identification with their professional roles. Importantly, the non-significant total effect should not be interpreted as “no effect.” Instead, it results from the cancelation of two opposing pathways. This suppression effect has both methodological and practical implications. Methodologically, it demonstrates that examining only total effects would mistakenly conclude that surface acting is irrelevant to professional identity. Practically, it suggests that interventions should not simply aim to reduce surface acting but rather focus on replenishing emotional resources to interrupt the negative indirect pathway.

Both DA and ENFE significantly and negatively predicted emotional exhaustion, thereby positively influencing professional identity through the mediating pathway of emotional exhaustion. From the perspective of COR theory (Hobfoll, 2001), DA can be understood as a resource investment strategy: individuals actively change their cognitive appraisals of situations to align internal feelings with external demands. Although this process requires effort, it can generate positive emotional improvements and help replenish psychological resources (Grandey, 2000; Gross and John, 2003), thereby reducing rather than increasing emotional exhaustion. In contrast, ENFE embodies the ideal state of person-job fit (Diefendorff et al., 2005), generating resource gains through positive emotional experiences and reinforcing interpersonal feedback (Humphrey et al., 2015; Yin et al., 2019). Notably, while the present findings align with some studies (Anomneze et al., 2016; Wei and Zhang, 2025), others have reported non-significant relationships between DA and emotional exhaustion (Wang et al., 2019; Ma et al., 2023). This inconsistency may be attributable to cultural differences between Eastern and Western contexts, with deep acting potentially being more adaptive in Eastern cultures (Wang et al., 2019). An alternative explanation concerns the distinctive characteristics of the pre-service teacher population. Being at a critical stage of professional identity formation, their deep acting practices, through the identity construction process of “becoming a teacher in action” (Deng et al., 2018), not only consume resources but also bring resource gains (e.g., professional accomplishment), thereby exhibiting a protective effect against emotional exhaustion.

These findings have significant practical implications. Emotional exhaustion represents a critical mediating pathway through which emotional labor influences professional identity. Consequently, the management of pre-service teachers’ emotional labor should not simplistically aim to “reduce” or “avoid” such experiences, but rather focus on establishing resource-replenishment mechanisms. For instance, interventions such as internship supervision, peer support, and mindfulness training could enhance their psychological resource resilience. This helps interrupt emotional exhaustion while harnessing the positive effects of emotional labor.

### 4.3 The moderating role of social and emotional competence

This study found that the mediating pathway through which emotional labor influences professional identity via emotional exhaustion was moderated by SEC only within the context of SA, supporting Hypothesis 3 for this specific dimension. Specifically, SEC buffered the positive effect of SA on emotional exhaustion, consequently weakening the mediating role of emotional exhaustion in the relationship between SA and professional identity. SA reduced professional identity by increasing emotional exhaustion only at low levels of SEC. As SEC increased, this negative indirect pathway gradually diminished. An alternative interpretation is that the buffering effect of SEC may be context-dependent. In collectivist cultures such as China, suppressing negative emotions to maintain interpersonal harmony is socially valued. The negative impact of surface acting might be generally lower, so the incremental benefit of high SEC could be more critical only when suppression conflicts with personal values. Cross-cultural differences in emotional labor have been documented in existing research and are also identified as one of the future development trends (Mastracci and Adams, 2019; Humphrey, 2023; Ntim et al., 2024).

It is worth noting that in the models of DA and ENFE, although the moderation effect in the first stage was significant, the moderated mediation index did not reach a significant level. The absence of a moderating effect for DA and ENFE may be explained by their lower intrinsic resource demands. DA already involves cognitive reappraisal, partially overlapping with SEC’s regulatory function, leaving less variance for SEC to moderate. ENFE requires minimal regulation, providing little opportunity for SEC to exert an additional buffering effect. Thus, SEC’s protective role is most evident in the context of SA.

The moderating effect of SEC observed in this study aligns theoretically with previous research examining the moderating roles of psychological capital and emotional intelligence in the outcomes of emotional labor (Chen et al., 2024; Liu and Li, 2026). This study makes several novel contributions to the literature. First, it is the first to incorporate SEC as a moderating variable in a mechanistic model of PSTs’ professional identity formation. Second, it advances the understanding of emotional labor by adopting a dimension-specific approach. This study separately examines SA, DA and ENFE, revealing that the buffering effect of SEC is significant only for SA. This indicates that SEC’s buffering role is specific to the high resource-depleting strategy of surface acting. Third, by integrating JD-R and COR theories, the study provides a comprehensive explanation of how emotional labor influences professional identity through the mediating pathway of emotional exhaustion and under what conditions this effect is attenuated. From a practical perspective, these results highlight the importance of integrating SEC training into teacher education curricula, with particular attention to helping pre-service teachers manage the negative effects of surface acting. Social–emotional learning interventions that enhance PSTs’ abilities to recognize, understand, and regulate emotions—both their own and others’—not only support their psychological well-being (Collie et al., 2024) but also serve as a “psychological immune” barrier for the healthy development of professional identity.

## 5 Implications

First, this study broadens the research perspective on emotional labor by shifting the focus from in-service to pre-service teachers. It reveals the “double-edged sword” effect of emotional labor during the formative stage of professional identity and its underlying mechanisms. This expansion enriches both the scope of the research subjects and the theoretical depth of studies on the consequences of emotional labor. Second, by integrating COR theory with JD-R theory, this study constructed and tested the mediating pathway from emotional labor to professional identity via emotional exhaustion. These mechanisms remain under-explored in previous research. Third, this study identified the moderating role of SEC as a key personal resource, offering a novel explanatory perspective for understanding why the consequences of emotional labor vary across individuals. Furthermore, it provides empirical support for the application of social–emotional learning theory in educational contexts.

This study has several important implications for pre-service teacher education and teacher education reform. First, teacher education institutions should acknowledge the dual role of emotional labor in pre-service teachers’ professional development. Emotional labor should neither be simplistically regarded as a “burden” to be reduced nor should its resource-depleting risks be overlooked. It is recommended that reflective workshops on emotional labor be incorporated into observation and internship programs, guiding pre-service teachers to construct positive meanings from their emotional labor experiences. Second, emotional exhaustion constitutes a critical mediating pathway through which emotional labor undermines professional identity. Therefore, establishing mechanisms for replenishing pre-service teachers’ psychological resources is of paramount importance. Practical strategies may include implementing regular supervision systems for internship mentors, establishing peer support groups, and offering mindfulness-based stress reduction training. Third, the malleability of SEC renders it an ideal intervention target for promoting both mental health and professional identity among pre-service teachers. It is recommended that social–emotional learning modules be systematically integrated into teacher education curricula to enhance pre-service teachers’ abilities in emotional awareness, regulation, and interpersonal communication. Such interventions would strengthen their “psychological immunity” against the negative effects of emotional labor at its source, thereby fostering higher levels of professional identity.

## 6 Limitations and future research directions

Several limitations of this study must be acknowledged. First, a cross-sectional design inherently restricts causal inference. While the proposed model is theoretically grounded, the directionality of effects remains uncertain. Future studies should adopt longitudinal or experimental designs to examine the dynamic mechanisms linking emotional labor to professional identity and to evaluate SEC training interventions. Second, all variables were measured using self-report questionnaires, which may have raised concerns regarding common method bias and social desirability effects. To address these concerns, we implemented both procedural remedies and statistical tests. However, the CFA marker variable approach was limited by the lack of pre-specified marker variables in the current study. Despite these constraints, procedural remedies and statistical tests confirmed that common method bias did not significantly threaten the validity of our findings. Future research could further strengthen methodological rigor by employing multi-method assessments, including supervising teacher evaluations, performance ratings, and physiological indicators. Third, the sample was restricted to pre-service teachers, which may limit the generalizability of the findings to in-service teachers or other occupational groups. The mechanisms by which emotional labor operates may differ between pre-service and in-service populations owing to variations in professional experience and contextual demands. Future research should compare these groups to identify potential similarities and differences in how emotional labor functions. Fourth, all participants in this study were recruited from six universities in Shaanxi Province, China. Although the sample covered different types of institutions, the geographic restriction to a single province may introduce bias when generalizing the findings to other regions or cultural contexts. Provinces in China differ in terms of economic development, cultural norms, and teacher preparation models. These factors may moderate the relationship between emotional labor and professional identity. Therefore, future research should expand the sampling scope to include multiple regions and cross-cultural settings to test the stability and generalizability of the proposed model. Finally, this study focused exclusively on the moderating role of SEC. Future investigations should explore the moderating effects of other individual factors (e.g., psychological resilience, self-evaluation) and contextual factors (e.g., school support, classroom climate) within the emotional labor-outcome framework. Such efforts would contribute to a more comprehensive understanding of the boundary conditions under which emotional labor influences professional identity.

## Data Availability

The raw data supporting the conclusions of this article will be made available by the authors, without undue reservation.
